# A validated severity score for haemorrhoids as an essential prerequisite for future haemorrhoid trials

**DOI:** 10.1007/s10151-019-01936-9

**Published:** 2019-02-06

**Authors:** M. J. Lee, J. Morgan, A. J. M. Watson, G. L. Jones, S. R. Brown

**Affiliations:** 1 Department of Surgery, Sheffield Teaching Hospitals, Northern General Hospital, Sheffield, UK; 2 Department of Psychology, School of Social Sciences, Leeds Beckett University, Leeds, UK; 3 Department of Colorectal Surgery, Raigmore Hospital, Inverness, UK

**Keywords:** Haemorrhoid, Patient-reported outcome, Psychometrics

## Abstract

**Background:**

There is a lack of standardised outcomes for haemorrhoidal disease making comparison between trials difficult. A need for a very well validated severity score is essential to facilitate meta-analysis of comparative studies, enabling evidence-based clinical practice.

**Methods:**

The Hubble trial provides a large cohort of patients with haemorrhoidal disease randomised to rubber band ligation (RBL) or haemorrhoidal artery ligation. The haemorrhoid severity score (HSS) was collected on each patient at baseline, 6 weeks and 1 year after intervention. This allows for the responsiveness of the HSS instrument to be examined and compared with a more specific instrument, the Vaizey incontinence score (also collected). Responsiveness was tested using four methods (effect size, standardised response means (SRM), significance of change, and responsiveness statistic).

**Results:**

The four tests of responsiveness demonstrated that the HSS was more responsive to changes in the patient’s health status following both of the interventions compared to the Vaizey questionnaire. For example, between baseline and 6 weeks, the RBL intervention effect size scores and SRM calculations indicated a non-significant small amount of change (0.20 and 0.16 respectively). However, using the HSS, the effect size and SRM demonstrated a large magnitude of change (1.12 and 1.01, respectively) which was significant. Similar results were observed at 1 year. Significance of change scores and the index of responsiveness were also higher for the HSS questionnaire than the Vaizey across both treatment modalities.

**Conclusions:**

The HSS is a highly responsive tool for the detection of changes in haemorrhoid symptoms. It should form an essential patient-reported outcome tool for future studies on haemorrhoidal disease.

## Introduction

Haemorrhoidal disease is a common condition, and can present with symptoms including bleeding, pain and anal leakage. There is a range of surgical interventions available including rubber band ligation (RBL), haemorrhoid artery ligation (HAL), stapled haemorrhoidopexy and excisional haemorrhoidectomy, some of which have been assessed in randomised trials [1]. Although there have been attempts to amalgamate these trials and produce guidance regarding optimal treatment pathways, all are subject to interpretation and on occasion guidelines differ substantially [2–4]. One of the major challenges in the comparison of these different studies is the lack of standardised outcomes. Research in haemorrhoidal disease is often confounded by the poly-symptomatic nature of the disease process. Clinician-reported outcomes in this setting show low levels of inter-rater agreement, making them unreliable [5].

In benign conditions, quality of life is an important outcome measure, but is frequently not reported. Where it is reported, generic quality of life tools are used that may not reflect specifically on haemorrhoidal disease [6]. Other studies may use outcomes that are specific solely to aspects of haemorrhoidal disease such as prolapse or incontinence, and fail to capture changes in other outcomes related to haemorrhoids [7]. A validated haemorrhoid-specific outcome tool, which takes into account both the poly-symptomatic nature of the disease along with the effect of these symptoms on quality of life, is required to be able to compare interventions and guide optimal therapy. Attempts have been made to produce such a tool using a multi-symptom approach, the haemorrhoid severity score (HSS) introduced by Nystrom [6]. Whilst reflecting the appropriate symptomatology, this scoring system has not gained wide acceptance due probably to a lack of robust validation. Others have developed this system and validated it (the Sodergren score) but validation was based on a very small sample of patients [8].

Responsiveness is an essential quality of any health-related quality of life (HRQoL) measure and refers to the ability of an instrument to detect change over time, if a true change in the patient’s health status has occurred before and after an intervention or treatment [9]. It also provides evidence of an instrument’s validity as it should confirm that anticipated responses arise in accordance with corresponding changes in health [10]. The responsiveness of an instrument is ideally evaluated using a therapy of known effectiveness, such as one evaluated in a clinical trial.

The aim of this study was to establish the responsiveness of the HSS for use in the evaluation of patients’ haemorrhoids and determine the suitability of the instrument as an outcome measure in this context.

## Materials and methods

As the responsiveness of an instrument is ideally evaluated using a therapy of known effectiveness [10, 11], this validation was planned as a secondary analysis of HuBBLe trial data (ISRCTN41394716) [11]. As part of this study, all patients had provided informed consent for their data to be used for analysis.

The Hubble trial was a multi-centre, parallel group randomised controlled trial with a 1:1 allocation ratio comparing HAL with RBL in adults aged 18 years or over with symptomatic second- or third-degree haemorrhoids. The primary outcome was defined as the proportion of patients with recurrent haemorrhoids at 12 months post-procedure, as derived from the patient’s self-reported assessment in combination with general practitioner and hospital records. Recurrence was defined using a simple question 12 months after randomisation [5]. ‘At the moment, do you feel your symptoms from your haemorrhoids are: (1) Cured or improved compared with before starting treatment; or, (2) Unchanged or worse compared with before starting treatment?’. Secondary endpoints assessed at baseline, 6 weeks and 12 months included: the haemorrhoid symptom severity score as well as the Vaizey incontinence score [12], a VAS pain score and health state utility based on the EuroQoL-5D. The study randomised 185 patients to HAL and 187 to RBL, and showed 1-year recurrence rates of 49% and 30%, respectively. Six-month questionnaire data including HSS were captured for 137 RBL and 144 HAL patients, and 1-year questionnaire data for 125 and 131, respectively.

### Statistical analysis

The HSS comprises five items. All items included in a domain are scored between 0 and 3 (0 indicating best and 3 worst health status). A total score is obtained by summing the answers to each item. Lower scores indicate better haemorrhoidal health.

The Vaizey incontinence score questionnaire is a seven-item measure shown to outperform others in detecting faecal incontinence [12]. The Vaizey consists of seven items, three of which ask about the frequency of incontinence on a 4-point scale ranging from 0 = Never to 4 = Daily, followed by a single item about the extent to which symptoms alter lifestyle (using the same 4-point scale). The final three items are concerned with the severity of incontinence using a dichotomous No/Yes response scale (No = 0, Yes = 2 for items five and six, and 4 for item seven). The Vaizey score is calculated by summing responses across the seven items. A lower score indicates less faecal incontinence (e.g. 0 = perfect continence, 24 = totally incontinent).

Numerous methods are available to determine the responsiveness of an instrument. As there is no gold standard approach, it has been recommended that multiple methods are employed [9, 10]. Four different statistical analyses were used to evaluate the responsiveness of the HSS, including: (i) effect size, (ii) standardised response means, (iii) significance of change, and the (iv) responsiveness statistic. All statistical analyses were performed using SPSS version 23 (IBM Corp, Armonk NY,USA).

### Effect size

The effect size (ES) is an estimation of the magnitude of change. It is calculated by measuring the difference between the means pre- and post-treatment, and dividing this value by the standard deviation of the pre-treatment score [13]. The changes in health status are translated into a standard unit of measurement to aid interpretation. Generally accepted ES values are 0.20 (Small), 0.50 (Moderate) and 0.80+ (Large) [14]. A small effect size implies that treatment has little influence on the health status of patients as measured by that specific questionnaire or domain.

### Standardised response mean

The standardised response mean (SRM) is similar to ES. However, to calculate the SRM the mean change in scores (i.e. between baseline and follow-up) is divided by the standard deviation of change in score [10].

### Significance of change

The mean changes in domain scores were calculated for patients based upon their answers to the anchor question in the primary outcome of the trial (whether they felt ‘(i) Cured or improved compared with before starting treatment; or, (ii) Unchanged or worse compared with before starting treatment?’). These data were collected at 6 weeks and 1 year post-treatment.

### Responsiveness statistic

The responsiveness statistic compares subjects who report improvement following intervention using the two questions above, with those who report no improvement. It is calculated by dividing the mean change in score for patients reporting improvement by the SD of scores from those who report no improvement [13]. A responsiveness statistic value ≥ 1 indicates that an instrument is highly responsive to change, and a value of between 0.20 and 1 indicates an acceptable level of responsiveness [14].

## Results

### Demographics from Hubble

The RBL group included 176 participants, 172 of whom underwent the intervention. This included 99 (56%) male participants and had a mean age of 49.0 years (S.D. 12.9). Grade II haemorrhoids were present in 115 (65%) participants, grade III in 60 (34%) and grade was missing for 1 participant. The recurrence rate at 1 year was 49% (87 cases).

The HAL group included 161 participants, 158 of whom underwent the intervention. This included 85 (53%) male participants and had a mean age of 48.5 years (SD 13.5). Grade II haemorrhoids were present in 92 (57%) participants, grade III in 68 (42%) and grade was missing for 1 participant. The recurrence rate at 1 year was 30% (48 cases).

### Haemorrhoid outcomes

Most patients felt ‘cured or improved’ following the interventions (81% at 6 weeks; 72% after 1 year) although some patients reported that they felt ‘worse or unchanged’ 6 weeks and 1 year after treatment. Ninety-two percent of HAL group patients felt cured or improved after 6 weeks compared to 71% of those in the RBL group. There was little difference between the intervention groups at 1 year. Again, 71% of the RBL stated that they felt cured or improved compared with 73% of the HAL group (representing a decrease in positive appraisals from the 6-week self-reported recurrence). Responses to the recurrence question are summarised in Table 1.

**Table 1:** Recurrence in RBL and HAL treatment groups at 6 weeks and 1 year post-interventions

Patient’s self-report recurrence	All patients	RBL	HAL
6 Weeks			
‘Cured or improved’	237 (81%)	106 (71%)	131 (92%)
‘Unchanged or Worse’	55 (19%)	43 (29%)	12 (8%)
Total	292 (100%)	149 (100%)	143 (100%)
1 Year			
‘ Cured or improved’	183 (72%)	91 (71%)	92 (73%)
‘Unchanged or Worse’	71 (28%)	37 (29%)	34 (27%)
Total	254 (100%)	128 (100%)	126 (100%)

RBL rubber band ligation, HAL haemorrhoidal artery ligation

### Effect size and SRM—from baseline to 6 weeks (Table 2)

**Table 2:** Mean scores, effect sizes, and significance of change (paired *t* test) between baseline and 6 weeks on the Vaizey and HSS for the two treatment groups and overall

	*N*	Mean baseline	SD	*N*	Mean 6 weeks	SD	Mean difference (SD)	Effect size	SRM	Paired *t* test
*HAL*										
Vaizey HAL	126	5.23	4.84	126	4.29	5.12	0.94 (5.90)	0.20	0.16	*p* = 0.75
HSS HAL	133	6.48	3.08	133	3.02	2.97	3.47 (3.44)	1.12	1.01	*p* < 0.001
*RBL*										
Vaizey RBL	102	5.70	5.23	102	3.79	4.55	1.90 (4.83)	0.36	0.39	*p* < 0.001
HSS RBL	111	6.35	3.08	111	4.05	3.41	2.31 (3.19)	0.75	0.72	*p* < 0.001
*All*										
Vaizey All	228	5.44	5.01	228	4.07	4.87	1.37 (5.46)	0.27	0.25	*p* < 0.001
HSS All	244	6.42	3.07	244	3.48	3.21	2.94 (3.37)	0.96	0.87	*p* < 0.001

RBL rubber band ligation, HAL haemorrhoidal artery ligation, HSS haemorrhoid severity score

Using the Vaizey score, in the HAL group mean scores from baseline to 6 weeks decreased indicating an improvement (5.23–4.29). However, these changes were non-significant (*p* = 0.075). Effect size and SRM calculations indicated a small amount of change (0.20 and 0.16, respectively). Using the HSS, a significant change was identified between scores at baseline and week 6 (6.48–3.02, *p* < 0.001). The effect size and SRM also demonstrated a large magnitude of change (1.12 and 1.01, respectively).

In the RBL group, using the Vaizey, mean scores between baseline and 6 weeks in the patient sample improved (5.70–3.79) which was significant (*p* < 0.001), with small to moderate effect size and SRMs (0.36 and 0.39, respectively). However, using the HSS, whilst a significant change was also observed (6.35–4.05, *p* < 0.001), the magnitude of change was greater as demonstrated by the effect size (0.75) and SRM (0.72).

### Effect size and SRM—from baseline to 1 year (Table 3)

**Table 3:** Mean scores, effect sizes, and significance of change (paired *t* test) between baseline and 1 year on the Vaizey and haemorrhoid severity scores for the two treatment groups and overall

	*N*	Mean baseline	SD	*N*	Mean 1 year	SD	Mean difference (SD)	Effect size	SRM	Paired *t* test
*HAL*										
Vaizey HAL	98	5.63	4.82	98	4.61	4.76	1.02 (4.91)	0.21	0.21	*p* = 0.042
HSS HAL	117	6.18	2.99	117	3.63	3.31	2.55 (3.34)	0.85	0.76	*p* < 0.001
*RBL*										
Vaizey RBL	94	4.84	4.49	94	3.60	4.38	1.25 (4.77)	0.28	0.26	*p* = 0.013
HSS RBL	104	6.03	3.19	104	3.62	3.31	2.41 (3.79)	0.76	0.64	*p* < 0.001
*All*										
Vaizey All	192	5.24	4.67	192	4.11	4.60	1.13 (4.83)	0.24	0.23	*p* = 0.001
HSS All	221	6.11	3.08	221	3.62	3.31	2.48 (3.55)	0.81	0.70	*p* < 0.001

RBL rubber band ligation, HAL haemorrhoidal artery ligation, HSS haemorrhoid severity score

Using the Vaizey score, for the HAL group mean scores from baseline to 1 year significantly decreased indicating an improvement (5.63–4.61, *p* = 0.04) but the effect size and SRM indicated only the smallest amount of change (0.21 and 0.21, respectively). Using the HSS, a significant change from baseline to 1 year was observed (6.18–3.63, *p* < 0.001). The effect size and SRM also demonstrated a large magnitude of change (0.85 and 0.76, respectively).

In the RBL group, using the Vaizey, mean scores between baseline and 1 year the patient sample improved (4.84–3.60) which was significant (*p* = 0.013), with small effect sizes and SRMs (0.28 and 0.26, respectively). However, using the HSS, whilst a significant change was also observed (6.03–3.62, *p* < 0.001), the magnitude of change was greater as demonstrated by the effect size (0.76) and SRM (0.64).

There was no significant difference in either group (HAL or RBL) from pre-randomisation score to baseline score (Table 4).

**Table 4:** Mean scores, effect sizes, and significance of change (paired *t* test) between pre-randomisation and baseline on the Vaizey and haemorrhoid severity scores for the two treatment groups combined

	*N*	Mean pre-randomisation	SD	*N*	Mean baseline	SD	Effect size	Paired *t* test
Vaizey	123	5.44	4.99	123	5.47	4.93	0.06	*p* = 0.875
HSS	127	6.35	3.32	127	6.39	3.23	0.014	*p* = 0.732

RBL rubber band ligation, HAL haemorrhoidal artery ligation, HSS haemorrhoid severity score

### Significance of change: cured after treatment

The effect size and significance of change scores between baseline and 6 weeks were derived for patients who had undergone HAL and RBL treatment, and reported themselves to be ‘cured or improved’ or ‘unchanged or worse’ at 6 weeks as based upon their own self-reported answers to the recurrence question (Table 5). For the patients who rated themselves as ‘cured or improved’ that received the HAL intervention, this change was only significant using the HSS questionnaire (*p* < 0.001). The effect size and SRM revealed the magnitude of change to be greater using this questionnaire (1.18 and 1.11 respectively) compared to the Vaizey Questionnaire which detected only a small amount of change (0.24 and 0.21, respectively).

**Table 5:** HAL mean scores, effect sizes, and significance of change (paired *t* test) between baseline and 6 weeks on the Vaizey and haemorrhoid severity scores for the two treatment groups for patients who reported themselves to be “cured or improved” and for patients who reported themselves to be “unchanged or worse” at 6 weeks

	*N*	Mean baseline (SD)	Mean 6 weeks (SD)	Mean difference	Effect size	SRM	95% CI	Paired *t* test
*Cured or improved*								
HAL								
Vaizey HAL	114	5.33 (5.01)	4.11 (5.10)	1.22 (5.94)	0.24	0.21	0.12–2.32	*p* = 0.30
HSS HAL	121	6.42 (3.17)	2.67 (2.60)	3.75 (3.38)	1.18	1.11	3.14–4.36	*p* < 0.001
RBL								
Vaizey RBL	74	5.26 (4.94)	3.27 (4.42)	1.97 (4.63)	0.40	0.43	0.91–3.06	*p* < 0.001
HSS RBL	79	5.94 (3.00)	3.01 (2.81)	2.92 (3.08)	0.97	0.95	2.23–3.61	*p* < 0.001
*Unchanged or Worse*								
HAL								
Vaizey HAL	10	4.20 (2.82)	6.40 (5.72)	− 2.20 (5.20)	− 0.78	− 0.42	From − 5.92 to 1.52	*p* > 0.05
HSS HAL	10	7.50 (1.58)	7.50 (3.87)	0.00 (2.58)	0.00	0.00	From − 1.84 to 1.84	*p* > 0.05
RBL								
Vaizey RBL	26	6.73 (6.02)	5.58 (4.60)	1.15 (5.21)	0.19	0.22	From − 0.95 to 3.26	*p* > 0.05
HSS RBL	30	7.50 (3.06)	6.97 (3.27)	0.53 (2.85)	0.17	0.19	From − 0.53 to 1.60	*p* > 0.05

RBL rubber band ligation, HAL haemorrhoidal artery ligation, HSS haemorrhoid severity score

For ‘cured or improved’ patients who underwent the RBL intervention, a similar trend was observed. Whilst the mean scores demonstrated an improvement in line with the self-report answers, the effect sizes and SRMs revealed only a moderate amount of change using the Vaizey (0.40 and 0.43) compared to the HSS which detected a large magnitude of change (0.97–0.95, respectively).

In patients who reported themselves to be unchanged or worse, no significant differences were observed for either the HAL or RBL treatment groups and the magnitude of change was small as indicated by the effect sizes and SRMs. In the HAL group, the mean scores on the Vaizey indicated the patient group had got worse (4.20–6.40) but the mean scores on the HSS revealed they had the stayed the same (7.50–7.50).

### Significance of change: cured after treatment (from baseline to 6 weeks—cured at 1 year)

The effect size and significance of change scores between baseline and 6 weeks were calculated as above for patients in either intervention group who self-reported that their condition was ‘cured or improved’ or ‘unchanged or worse’ at 1 year (Table 6). For the ‘cured or improved’ patients in the HAL intervention group, this change was again only significant using the HSS Questionnaire who rated themselves as ‘cured or improved’ (*p* < 0.001). The effect size and SRM revealed the magnitude of change to be greater using this questionnaire (1.03 and 0.99, respectively) compared to the Vaizey Questionnaire which detected only a small amount of change (0.16 and 0.13, respectively).

**Table 6:** Mean scores, effect sizes, and significance of change (paired *t* test) between baseline and 6 weeks on the Vaizey and HSS for the two treatment groups for patients who reported themselves to be “cured or improved” and for patients who reported themselves to be “unchanged or worse” at 1 year

	*N*	Mean baseline (SD)		Mean 6 weeks (SD)	Mean difference	Effect size	SRM	95% CI	Paired *t* test
*Cured or improved*									
HAL									
Vaizey HAL	73	5.52 (5.08)		4.71 (5.57)	0.81 (6.03)	0.16	0.13	From − 0.60 to 2.21	*p* > 0.05
HSS HAL	78	5.78 (3.21)		2.46 (2.51)	3.32 (3.36)	1.03	0.99	2.56–4.08	*p* < 0.001
RBL									
Vaizey RBL	55	4.85 (4.65)		3.55 (4.21)	1.31 (4.69)	0.28	0.28	0.04–2.58	*p* = 0.04
HSS RBL	60	6.40 (3.22)		3.37 (3.01)	3.03 (3.01)	0.94	1.01	2.26–3.81	*p* < 0.001
*Unchanged or worse*									
HAL									
Vaizey HAL	27	5.56 (4.78)		4.19 (4.49)	1.37 (5.92)	0.29	0.23	From − 0.97 to 3.71	*p* > 0.05
HSS HAL	28	7.29 (2.54)		4.43 (3.05)	2.86 (3.09)	1.13	0.93	1.66–4.05	*p* < 0.001
RBL									
Vaizey RBL	27	5.85 (5.80)		4.04 (5.09)	1.82 (4.58)	0.31	0.40	0.00–3.63	*p* = 0.05
HSS RBL	28	5.93 (3.25)		4.93 (3.63)	1.00 (2.84)	0.31	0.35	From − 0.10 to 2.10	*p* > 0.05

RBL rubber band ligation, HAL haemorrhoidal artery ligation, HSS haemorrhoid severity score

This finding was replicated for RBL patients self-reporting ‘cured or improved’. Whilst the mean scores demonstrated an improvement in line with the self-report answers, the effect sizes and SRMs revealed only a small to moderate amount of change using the Vaizey (0.28 and 0.28) compared to the HSS which detected a large magnitude of change (0.94–1.01, respectively).

Due to the incongruence between the timing of patient ratings of changes in health status (cured or not at 1 year) and the significance of change period (from baseline to 6 weeks), patients in both intervention groups who reported ‘unchanged or worse’ condition after 1 year showed a decrease in Vaizey and HSS scores at 6 weeks (i.e. an improvement). This change in the HAL group was only significant (*p* < 0.001) when using the HSS and not the Vaizey. The effect size and SRM suggest a large magnitude of change (1.13 and 0.93, respectively).

For patients who reported themselves to be ‘unchanged or worse’ in the RBL intervention group, effect sizes and SRM were very similar, and small to moderate, for the Vaizey (0.31 and 0.40, respectively) and HSS (0.31 and 0.35, respectively). The change detected by the Vaizey was significant (*p* = 0.05).

### Significance of change: cured after treatment (from baseline to 1 year—cured at 1 year) (Table 7)

**Table 7:** Mean scores, effect sizes, and significance of change (paired *t* test) between baseline and 1 year on the Vaizey and HSS for the two treatment groups for patients who reported themselves to be “cured or improved” and for patients who reported themselves to be “unchanged or worse” at 1 year

	*N*	Mean baseline (SD)	Mean 1 year (SD)	Mean difference	Effect size	SRM	95% CI	Paired *t* test
*Cured or improved*								
HAL								
Vaizey HAL	71	5.35 (4.87)	3.82 (4.44)	1.54 (4.81)	0.32	0.32	0.40–2.67	*p* < 0.01
HSS HAL	84	5.69 (3.05)	2.21 (2.12)	3.48 (3.08)	1.14	1.13	2.81–4.14	*p* < 0.001
RBL								
Vaizey RBL	68	4.85 (4.36)	3.40 (4.23)	1.46 (4.76)	0.33	0.31	0.31–2.61	*p* < 0.01
HSS RBL	74	6.11 (3.22)	2.57 (2.22)	3.54 (3.62)	1.01	0.98	2.70–4.38	*p* < 0.001
*Unchanged or worse*								
HAL								
Vaizey HAL	27	6.37 (4.71)	6.70 (5.02)	− 0.33 (4.99)	− 0.07	− 0.07	− 2.31–1.64	*p* > 0.05
HSS HAL	33	7.42 (2.45)	7.24 (3.07)	0.18 (2.79)	0.07	0.06	− 0.81–1.17	*p* > 0.05
RBL								
Vaizey RBL	26	4.81 (4.92)	4.12 (4.80)	0.69 (4.86)	0.14	0.14	− 1.27–2.66	*p* > 0.05
HSS RBL	30	5.83 (3.16)	6.20 (4.11)	− 0.37 (2.59)	0.12	− 0.14	From − 1.34 to 0.60	*p* > 0.05

RBL rubber band ligation, HAL haemorrhoidal artery ligation, HSS haemorrhoid severity score

The effect size and significance of change scores between baseline and 1 year were also calculated for patients in either intervention group who self-reported that their condition was ‘cured or improved’ or ‘unchanged or worse’ at 1 year (Table 7). For the patients who rated themselves as ‘cured or improved’ who underwent the HAL intervention, this change was significant using both the Vaizey (*p* < 0.01) and HSS (*p* < 0.001) Questionnaires. Effect size and SRM revealed the magnitude of change to be greater using the HSS (1.14 and 1.13, respectively) compared to the Vaizey Questionnaire which detected only a small to moderate amount of change (0.32 and 0.32 respectively).

For ‘cured or improved’ patients who underwent the RBL intervention, a similar trend was observed. Whilst the mean scores demonstrated an improvement in line with the self-report answers, the effect sizes and SRMs revealed only a small to moderate amount of change using the Vaizey (0.33 and 0.31) compared to the HSS which detected a large magnitude of change (1.01–0.98, respectively).

The findings suggest that patients in both intervention groups who at 1 year rated themselves as ‘unchanged or worse’ at 1 year may have experienced a short-lived improvement post-intervention (from baseline to 6 weeks). The HSS but not Vaizey showed a significant large magnitude change for the HAL intervention group, while both HSS and the Vaizey detected small improvements for RBL patients. This supposition is supported by the significance of change results between baseline and 1 year for patients cured or not also at 1 year (Table 7) showing no significant changes for ‘unchanged or worse’ patients.

For patients who reported themselves to be ‘unchanged or worse’, no significant differences were observed for either the HAL or RBL treatment groups and the magnitude of change was very small as indicated by the effect sizes and SRMs.

### Responsiveness statistic

The responsiveness statistic was calculated for the Vaizey and HSS for both the HAL and RBL treatment groups (Table 8**)**. Values for the responsiveness statistic using the Vaizey ranged from 0.23 to 0.38. However, this was much higher using the HSS Questionnaire, where the responsiveness values ranged between 1.02 and 1.45, indicating that this measure was highly responsive to change.

**Table 8:** Responsiveness statistic for the treatment groups at 6 weeks and 1 year

Measure	Mean score change (SD)		Responsiveness statistic
	“Cured or improved”	“Worse or not changed”	
*HAL*			
Vaizey (baseline–6 weeks)	1.22 (5.94)	− 2.20 (5.20)	0.23
HSS (baseline–6 weeks)	3.75 (3.38)	0.00 (2.58)	1.45
Vaizey (baseline–1 year)	1.54 (4.81)	− 0.33 (4.99)	0.31
HSS (baseline–1 year)	3.48 (3.08)	0.18 (2.79)	1.25
*RBL*			
Vaizey (baseline–6 weeks)	1.97 (4.63)	1.15 (5.21)	0.38
HSS (baseline–6 weeks)	2.92 (3.08)	0.53 (2.85)	1.02
Vaizey (baseline–1 year)	1.46 (4.76)	0.69 (4.86)	0.30
HSS (baseline–1 year)	3.54 (3.62)	− 0.37 (2.59)	1.37

HSS haemorrhoid severity score

## Discussion

This study was a planned secondary analysis of trial data undertaken to determine the responsiveness of the HSS in the context of RBL and HAL as treatments for haemorrhoids. The results indicate that the HSS is more responsive to change in patients’ health status than the Vaizey scale for both procedures as measured by effect sizes, SRMs, significance of change scores and the index of responsiveness. The instrument, therefore, appears to be suitable for use as an outcome measure in this context.

This is perhaps not surprising. The Vaizey scale was developed to assess the severity of faecal incontinence and not haemorrhoids specifically [12], and so it is reassuring that the results of the responsiveness analyses confirmed that the HSS measure was more sensitive in detecting changes in patients’ health status following treatments for haemorrhoids than the Vaizey measure. This adds support to the validity of the HSS measure although more tests to determine the validity of the measure are needed. The validity of the HSS is further supported as the findings of the measure match the clinical experience in the use of HAL in the HuBBLe trial [11].

There is a wealth of literature about the treatment of haemorrhoids. Whilst many publications are case series or reviews, there are over 400 randomised controlled trials and over 40 meta-analyses. One would, therefore, assume that the correct therapy for haemorrhoids should be well defined and yet this is not the case. There are a number of shortcomings in this ‘high-quality literature’ making it difficult to determine what is the optimal treatment [15]. Significant variations in recent guideline recommendations are testament to how the evidence can be interpreted differently [2–5]. One major issue with trial data interpretation is the lack of standardisation of outcome measures. A systematic review by van Tol details over 59 different outcome measures that have been used. They have shown varied definitions of outcomes; in many cases, outcomes were not defined at all [15].

There has been a previous attempt at validating a haemorrhoid severity score [7, 8]. However, this analysis was based on a very small cohort. External validity was demonstrated by showing those with higher scores were more likely to undergo surgery. There was no demonstration of change with time or after treatment. In contrast, we have demonstrated in two large cohorts from a carefully designed randomised trial that the HSS is highly responsive to intervention and represents a much more robustly validated tool for the assessment of haemorrhoidal treatment that will facilitate comparative studies and allow more meaningful synthesis of research data.

More recently, a new patient-reported outcome measure has been developed to capture the burden associated with haemorrhoidal disease and anal fissures upon quality of life (HEMO-FISS-QoL) [7]. Whilst some psychometric properties have been determined (e.g. acceptability, construct validity and reliability), the responsiveness of this measure is yet to be determined. The measure is also not specific to the haemorrhoid population as it is also designed to report outcomes following treatment of anal fissures. This may affect its performance when compared to the HSS.

### Strengths and limitations

This analysis does have some limitations. We have only captured quantitative data. Additional qualitative information on participant interpretation of questions as well as ease of completion would have been informative. However, the form, consisting of five simple questions, is by no means onerous. Another limitation is the fact that the HSS does not include a global satisfaction domain. Just because the HSS score is improved, this does not indicate that the patient is satisfied with the outcome and the intervention can be classified as a ‘success’. An example would be a patient who complains of bleeding but also has prolapse. The intervention cures the bleeding but fails to cure the prolapse. Is the patient ‘cured’? Nystrom recommended classifying cure as an HSS score of 0 or 1 [6]. This is perhaps inadequate to capture those who still have symptoms but who are adequately improved so as to be content with the outcome. We, therefore, recommend including an additional global satisfaction score in any haemorrhoidal disease research, such as that suggested by Shanmugan *et al* in a Cochrane review [16].

### Implications for future research

To be able to combine comparative trials in a scientifically valid way, a core outcome set of outcomes must be developed. Van Tol and colleagues are seeking to develop these core outcomes via a Delphi exercise [17]. It is very likely that the patient-reported outcome domain of this core outcome set will include the components contained within the HSS. Given the poly-symptomatic nature of haemorrhoidal disease, a way of combining these patient-reported outcomes in a validated and responsive format is required. Our analysis clearly shows that the HSS meets these requirements.

## Conclusions

The HSS is a highly responsive tool for the detection of changes in haemorrhoid symptoms, and should be recommended for use as a patient-reported outcome measure in all future clinical trials investigating haemorrhoidal disease.

## References

[1] Simillis C, Thoukididou SN, Slesser AAP (2015). Systematic review and network meta-analysis comparing clinical outcomes and effectiveness of surgical treatments for haemorrhoids. Br J Surg.

[2] Davis BR, Lee-Kong SA, Migaly J (2018). The American Society of Colon and Rectal Surgeons Clinical Practice Guidelines for the Management of Hemorrhoids. Dis Colon Rectum.

[3] Trompetto M, Clerico G, Cocorullo GF (2015). Evaluation and management of hemorrhoids: Italian society of colorectal surgery (SICCR) consensus statement. Tech Coloproctol.

[4] Agarwal N, Singh K, Sheikh (2017). Executive summary—the association of colon & rectal surgeons of India (ACRSI) practice guidelines for the management of haemorrhoids-2016. Indian J Surg.

[5] Brown S, Tiernan J, Biggs K (2016). The HubBLe Trial: haemorrhoidal artery ligation (HAL) versus rubber band ligation (RBL) for symptomatic second- and third-degree haemorrhoids: a multicentre randomised controlled trial and health-economic evaluation. Health Technol Assess.

[6] Nyström P-O, Qvist N, Raahave D (2010). Randomized clinical trial of symptom control after stapled anopexy or diathermy excision for haemorrhoid prolapse. Br J Surg.

[7] Abramowitz L, Bouchard D, Siproudhis L (2018). Psychometric properties of a questionnaire (HEMO-FISS-QoL) to evaluate the burden associated with haemorrhoidal disease and anal fissures. Colorectal Dis.

[8] Pucher PH, Qurashi M, Howell A-M (2015). Development and validation of a symptom-based severity score for haemorrhoidal disease: the Sodergren score. Colorectal Dis.

[9] Walters SJ, Brazier JE (2003). What is the relationship between the minimally important difference and health state utility values? The case of the SF-6D. Health Qual Life Outcomes.

[10] Guyatt GH, Osoba D, Wu AW (2002). Methods to explain the clinical significance of health status measures. Mayo Clin Proc.

[11] Brown SR, Tiernan JP, Watson AJM (2016). Haemorrhoidal artery ligation versus rubber band ligation for the management of symptomatic second-degree and third-degree haemorrhoids (HubBLe): a multicentre, open-label, randomised controlled trial. Lancet.

[12] Vaizey CJ, Carapeti E, Cahill JA, Kamm MA (1999). Prospective comparison of faecal incontinence grading systems. Gut.

[13] Lydick E, Epstein RS (1993). Interpretation of quality of life changes. Qual Life Res.

[14] Lohr KN, Aaronson NK, Alonso J (1996). Evaluating quality-of-life and health status instruments: development of scientific review criteria. Clin Ther.

[15] van Tol RR, van Zwietering E, Kleijnen J (2018). Towards a core outcome set for hemorrhoidal disease-a systematic review of outcomes reported in literature. Int J Colorectal Dis.

[16] The Cochrane Collaboration (1996). Rubber band ligation versus excisional haemorrhoidectomy for haemorrhoids. Cochrane database of systematic reviews.

[17] van Tol RR, Melenhorst J, Dirksen CD, Stassen LPS, Breukink SO (2017). Protocol for the development of a CoreOutcome Set (COS) for hemorrhoidal disease: an international Delphi study. Int J Colorectal Dis.

